# Report Made to the Philomathic Society of Paris, by Dr. Edwards, Sen. and M. Jules Cloquet, on a Memoir Transmitted to the Society by Dr. Barry, and Entitled "on the Application of the Barometer to the Study of the Circulation of the Blood, and of Respiration in the Vertebrated Animals."

**Published:** 1827-12

**Authors:** 


### CIRCULATION OF THE BLOOD.

*Report made to the Philomathic Society of Paris, by* Dr. Edwards, sen. *and* M. Jules Cloquet, *on a Memoir transmitted to the Society by* Dr. Barry, *and entitled "On the Application of the Barometer to the Study of the Circulation of the Blood, and of Respiration in the Vertebrated Animals."* (From a Correspondent.)

The Society has charged us, M. Jules Cloquet and myself, to report upon a memoir written by Dr. Barry, and entitled as above. As the reading of the memoir was anterior to the nomination of its author to be one of our corresponding members, we cannot omit the report which we have been desired to make; and we are the less inclined to this omission, because we are convinced that the Society will listen with interest to the results of the experiments which Dr. Barry has repeated.

As these experiments are founded on physical principles, we entreated MM. Ampère and Savart to be present. Though we could not entertain any doubts as to the results of the experiments already performed by Dr. Barry, before MM.

Cuvier and Dumeril,—viz. that the pressure of the atmosphere contributes to the progression of the venous blood towards the heart during inspiration; yet we were anxious to ascertain, at least approximately, to what extent this cause operates. For this purpose Dr. Barry repeated the following experiments:

He introduced one end of a bent glass tube under the posterior extremity of the sternum of a pigeon placed upon his back, and pushed it as far as the region of the heart. The other extremity of the tube was immersed in a vessel containing a coloured liquid. When the animal inspired, the liquid rose in the tube, and descended when he expired. M. Savart, who measured the height of the ascent of the liquid, estimated it at two centimetres. As the tendency to vacuum takes place during inspiration, it follows (abstraction being made of all unforeseen disturbing causes,) that, according as the entrance of the air may be more or less free, the afflux of blood to the thoracic cavity will be in an inverse proportion, —that is to say, the more freely the air enters into the lungs, the less blood will arrive in the cavity when the tendency to vacuum is established, and *vice versâ*. We wished to see the proof of this by experiment, because, in the complicated animal organisation, the results, which may be rigorously inferred from certain well-attested facts, are not always produced by the experiments undertaken to verify them, and because the confirmation of this consequence would form the basis of an important application to pathology, as we shall presently see. Whilst the animal was circumstanced as I have already stated, Dr. Barry slightly compressed the trachea, and the liquid rose higher than in the other experiment, when the air had a free entrance. As the pressure was increased, the liquid rose higher and higher, and, when the trachea was entirely closed, the liquid rose so as to fill the whole tube, which was six inches long. This experiment shows clearly the truth of the general principle, and also the important part which the pressure of the atmosphere plays in pathological cases, when the entrance of the air is rendered more difficult by partial obstruction, or narrowing of the air-passages.

A modification of this experiment presented to us a curious phenomenon, which we shall state *en passant*, though it has no reference to the principles which Dr. Barry endeavours to establish in his memoir.

Leaving one end of the tube in the thorax of the animal, Dr. Barry removed the other end from the glass containing the coloured liquid, that it might communicate freely with the atmosphere; at the same time he tied the trachea, so as to prevent completely the entrance of air into the lungs. Air, however, penetrated into the thorax by the end of the tube which communicated with the atmosphere, but did not penetrate the lungs. Yet the animal, instead of dying almost suddenly, (as happens when the trachea is closed without employing a tube, as in this experiment,) continued to live without any convulsive movement, and apparently as if he still respired by the trachea. We remarked, however, that the capillaries had not their natural colour, and that the skin inclined to violet. The air which entered the thorax was in contact with the surface of the lungs, and produced in the blood the changes necessary for the support of life, but in a proportion less than in a state of health.

The other series of facts which we had to verify, referred to the influence of atmospheric pressure on the motion of the blood in the cold-blooded vertebrata. The individual made use of was a frog. The dispositions for the experiment were the same as in the case of a pigeon: that is to say, a bent tube of glass was introduced under the posterior extremity of the sternum of the animal placed on his back. The tube was pushed as far as the anterior region of the heart, whilst the outer extremity was immersed in a coloured liquid. During the respiratory movements of the frog, there was a motion of ascent and descent of the liquid in the tube, of about two millimetres.

It was not thought necessary that Dr. Barry should repeat the experiment upon the snake, because the mechanism of respiration in the ophidian class very much resembles the manner in which birds produce a vacuum during inspiration. Experiments similar to those repeated upon the pigeon would necessarily afford analogous results. He wished, however, that one of us should witness the experiment: it was repeated, and the results agreed with the deduction we had formed from the experiment upon the pigeon.

We did not repeat the experiment which goes to prove that there is, in the pericardium of fishes, a tendency to a vacuum caused by the locomotion of the heart, because, according to the details of this experiment, the action is so weak that it is marked only by a change of curve on the surface of the mercurial column in the barometer.

Dr. Barry showed to one of us experiments which tend to prove that there is another force accessory to that of the heart which contributes to the centrifugal, or outward, motion of the blood in fishes. As these experiments are not stated in the memoir, the object of this report, we purpose to communicate them to the Society upon another occasion.

Dr. Barry requested one of us to witness an experiment intended to ascertain the extent to which the inspiratory efforts of a middle-sized dog can bring down the mercurial column in a barometer. For this purpose, he fitted one end of a flexible tube into the thoracic opening of the divided trachea of a dog, the other end into an opening made in the reservoir of a barometer. During the strongest inspirations, the barometric column descended about five inches, and remained there for a moment.

*Note.*—The author being a member of the Society when the report was read, no opinion could be given by the reporters as to the merits of the memoir. The report was adopted, and the memoir was ordered to be printed with those of the Society.

---

